# Long-term overall survival and prognostic score predicting survival: the IMPACT study in precision medicine

**DOI:** 10.1186/s13045-019-0835-1

**Published:** 2019-12-30

**Authors:** Apostolia-Maria Tsimberidou, David S. Hong, Jennifer J. Wheler, Gerald S. Falchook, Filip Janku, Aung Naing, Siqing Fu, Sarina Piha-Paul, Carrie Cartwright, Russell R. Broaddus, Graciela M. Nogueras Gonzalez, Patrick Hwu, Razelle Kurzrock

**Affiliations:** 1 Department of Investigational Cancer Therapeutics, Phase I Clinical Trials Program, Unit 455, The University of Texas MD Anderson Cancer Center, 1515 Holcombe Boulevard, Houston, TX 77030 USA; 2 Current Address: TScan Therapeutics, Waltham, USA; 3 Current Address: Sarah Cannon Research Institute, Nashville, USA; 4 Department of Pathology, The University of Texas MD Anderson Cancer Center, Houston, USA; 5 Department of Biostatistics, The University of Texas MD Anderson Cancer Center, Houston, USA; 6 Department of Melanoma Medical Oncology, The University of Texas MD Anderson Cancer Center, Houston, USA; 7 Current Address: Moores Cancer Center—University of California San Diego, San Diego, USA

**Keywords:** Personalized medicine, Phase I, Clinical trials, Targeted therapy, Genomic profiling, Precision oncology

## Abstract

**Background:**

In 2007, we initiated IMPACT, a precision medicine program for patients referred for participation in early-phase clinical trials. We assessed the correlation of factors, including genomically matched therapy, with overall survival (OS).

**Patients and methods:**

We performed molecular profiling (Clinical Laboratory Improvement Amendments) (genes ≤ 182) for patients with lethal/refractory advanced cancers referred to the Phase 1 Clinical Trials Program. Matched therapy, if available, was selected on the basis of genomics. Clinical trials varied over time and included investigational drugs against various targets (single agents or combinations). Patients were followed up for up to 10 years.

**Results:**

Of 3487 patients who underwent tumor molecular profiling, 1307 (37.5%) had ≥ 1 alteration and received therapy (matched, 711; unmatched, 596; median age, 57 years; 39% men). Most common tumors were gastrointestinal, gynecologic, breast, melanoma, and lung. Objective response rates were: matched 16.4%, unmatched 5.4% (*p* < .0001); objective response plus stable disease ≥ 6 months rates were: matched 35.3% and unmatched 20.3%, (*p* < .001). Respective median progression-free survival: 4.0 and 2.8 months (*p* < .0001); OS, 9.3 and 7.3 months; 3-year, 15% versus 7%; 10-year, 6% vs. 1% (*p* < .0001). Independent factors associated with shorter OS (multivariate analysis) were performance status > 1 (*p* < .001), liver metastases (*p* < .001), lactate dehydrogenase levels > upper limit of normal (*p* < .001), PI3K/AKT/mTOR pathway alterations (*p* < .001), and non-matched therapy (*p* < .001). The five independent factors predicting shorter OS were used to design a prognostic score.

**Conclusions:**

Matched targeted therapy was an independent factor predicting longer OS. A score to predict an individual patient’s risk of death is proposed.

**Trial registration:**

ClinicalTrials.gov, NCT00851032, date of registration February 25, 2009.

## Introduction

Over the past 10–15 years, as targeted agents entered the phase I clinical trial arena, it became evident that response rates were exceedingly low when these agents were applied to unselected patient populations. In contrast, the remarkable improvement in overall survival (OS) of patients with newly diagnosed Bcr-Abl-positive chronic myeloid leukemia (CML) treated with imatinib (a potent inhibitor of the aberrant Bcr-Abl tyrosine kinase) exemplified the benefit of targeted therapeutics. It was initially thought that matching targeted agents with cognate molecular alterations would not be effective in solid tumors because they are too heterogeneous and complex, but in 2007, we began the IMPACT (Initiative for Molecular Profiling and Advanced Cancer Therapy) study, a personalized, precision medicine program for patients referred to the Phase I Clinical Trials Program at The University of Texas MD Anderson Cancer Center. Precision medicine deploys conventional and emerging concepts of the genetic and environmental bases of disease to tailor prevention and treatment strategies to the individual [1] and incorporates patient molecular profiles into the treatment selection process [2]. The objective of IMPACT was to use genomics to optimize the selection of targeted drugs for patients being considered for phase I clinical trials. The study was designed on the basis of (a) the rapid emergence of powerful technologies that identify molecular aberrations, (b) the entry into the clinic of multiple drugs with well-defined molecular targets, and (c) the success of targeted therapeutics such as imatinib in CML [3].

We previously reported the preliminary results of the IMPACT trial, a validation and landmark analysis, and a subsequent patient group analysis [4–6]. Herein, we report on the long-term follow-up results of consecutive patients who had Clinical Laboratory Improvement Amendments (CLIA)-certified molecular profiling prior to treatment in our Phase I Clinical Trials Program. We analyzed patient outcomes according to the molecular pathway targeted and performed multivariate analyses for outcomes. A prognostic score for OS was developed, taking into consideration molecular pathways.

## Patients and methods

### Patients

Consecutive patients who were referred to our Phase I Clinical Trials Program from September 2007 to December 2013 and for whom molecular analysis was ordered were included. The methodology has been previously described [4]. Briefly, patients with advanced or metastatic cancer for whom the standard-of-care therapy had been exhausted or no Food and Drug Administration (FDA)-approved therapy was available for their indication were considered for participation in phase I clinical trials.

Patients with targetable tumor alterations were treated on clinical trials with matched therapy, when available. If matched therapy was unavailable, they received treatment on protocols with non-matched therapy. Clinical trials varied over time and included first-in-human investigational agents against various targets, drugs approved by the FDA for a specific alteration outside their labeled indication, or combinations of targeted agents with cytotoxics, cytokines, anti-vascular endothelial growth factor (EGF), or other agents. Assignment to a clinical trial was determined by the treating physicians and/or after discussion at a multidisciplinary conference. Treatment was selected on the basis of the patient’s tumor markers, diagnosis, prior response to therapy, and previous toxic effects. Patients had to meet the eligibility criteria, and insurance had to approve coverage of the cost.

All patients provided written informed consent stating that they were aware of the experimental nature of the specific phase I study in which they participated. Clinical trials and analyses were conducted with the approval of and in accordance with the guidelines of the MD Anderson Cancer Center Institutional Review Board. The trial was registered at www.clinicaltrials.gov (NCT00851032).

### Matched therapy

Patients were treated with matched therapy if they had an “actionable” molecular alteration, if a clinical trial was available, and if they agreed to comply with study requirements. Clinical trials with matched targeted therapy were not always available because of the study design (“3+3”, limited availability in multi-institutional studies) or, more importantly, because of the protocol exclusion/eligibility criteria. Clinical trials were sponsored by pharmaceutical companies or they were investigator-initiated trials. In general, these studies were targeting a specific marker regardless of the tumor type.

The agents studied included those targeting PIK3CA, mTOR, BRAF, MEK, multikinases, KIT, EGFR, and RET. Many of the targeted agents had multikinase inhibitory activity, and all were known to inhibit a molecular aberration at low nmol/L concentrations. PIK3CA mutations and PTEN loss could be targeted by PI3K, AKT, or mTOR inhibitors, as AKT and mTOR are downstream of activated PIK3CA and both PIK3CA mutations and PTEN loss activate PI3K. GNAQ, RAS, and BRAF mutations could be targeted by MEK inhibitors. BRAF mutations were also targeted by BRAF inhibitors. Other aberrations, such as RET, EGFR, KIT, and MET mutations, were targeted by drugs inhibiting the respective activated kinase. EGF receptor (EGFR) was targeted by anti-EGFR antibodies. As results of clinical trials became available, certain tumor types with adverse outcomes were excluded. For instance, patients with colorectal cancer bearing a BRAF V600E mutation were excluded from clinical trials with a BRAF inhibitor when data showing adverse outcomes associated with this approach in this tumor type became available.

### Analysis of molecular aberrations

Molecular profiling was performed in CLIA-certified molecular diagnostics laboratories [4]. The number of genes analyzed (up to 182 genes per patient) depended on the date of testing and tumor tissue available. Molecular alterations were originally categorized as follows: PI3K/AKT/mTOR pathway, MAPK signaling, tyrosine kinases, hormone pathway, and other (DNA repair pathway, cell cycle-associated genes, and TP53/tumor suppressor/apoptosis-associated genes) (Additional file 1: Table S1). Due to the small numbers of patients in some subsets, only these categories were used.

### Endpoints and statistical methods

Statistical analysis was performed by our biostatistician GMNG using Stata/SE version 15.1 statistical software (Stata Corp., College Station, TX). The analysis was retrospective and exploratory, but the patients were matched prospectively. Tumor response was assessed using the Response Evaluation Criteria in Solid Tumors (RECIST) [7, 8]. OS was measured from initiation of participation in the phase I trial until death or last follow-up. Progression-free survival (PFS) was measured from the first day of treatment on a clinical trial until the date of disease progression or death, whichever came first. Treatment was discontinued if there was evidence of disease progression by RECIST or toxicity or if the patient withdrew consent.

Patients’ characteristics were analyzed using descriptive statistics. Univariate and multivariate logistic regression models were used to determine the association between response to therapy and patients’ characteristics. Survival and hazard functions were estimated using the Kaplan-Meier method, and survival between groups was compared using the 2-sided log-rank test. Characteristics that were statistically significant in the univariate analysis were included in the multivariate analysis. The multivariate Cox proportional hazards regression model was used to adjust for risk factors related to OS and PFS.

Independent factors predicting OS in multivariate analysis were used to develop a prognostic score (Cox model; level of significance, *p* < 0.05). Then, we performed multivariate analyses to develop the model using a training set (70% of patients) and to test the model using a validation set (30% of patients). The estimated coefficients from the final Cox model were used to assign a score to each factor.

## Results

### Patient characteristics

**Fig. 1:**
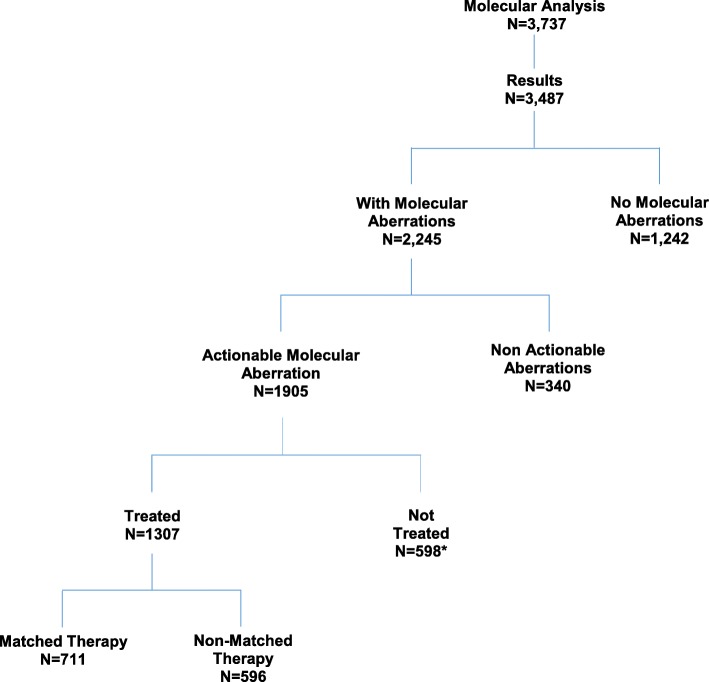
CONSORT diagram. *Overall, 598 patients with molecular aberrations did not receive treatment in our program for the following reasons: preference to be treated elsewhere or declined Phase I treatment (*n* = 230, 38.5%), ineligibility (*n* = 177, 29.6%), treated after the cut-off date of the period of analysis (*n* = 62; 10.4%), worsening performance status (*n* = 57; 9.5%), received regional therapy (*n* = 31, 5.2%), lost to follow-up (*n* = 23, 3.8%), or insurance issues (*n* = 18; 3%)

Tumor molecular profiling was ordered for 3737 consecutive patients (Table 1) who were referred for treatment, and 3487 patients had adequate tissue for analysis. Overall, 1307 (37.5%) patients had ≥ 1 aberration and received treatment (Fig. 1). The median patient age was 57 years (range, 16–86); 39% were men. The most common tumor types were gastrointestinal, 24.2%; gynecological, 19.4%; breast, 13.5%; melanoma, 11.9%; and lung, 8.7%. The median number of prior therapies was 4 (range, 0–16); and 2.8% of patients were previously untreated. The numbers of patients with the most common aberrations were as follows: ER overexpression, 346 patients; *KRAS* mutation, 307; *TP53* mutation, 223; *PIK3CA* mutation, 210; *BRAF* mutation, 189; PTEN loss or mutation, 184; PR overexpression, 167; MET mutation or amplification, 72; *EGFR* mutation, 71; *NRAS* mutation, 66; HER2 amplification, 61; and *CKIT* mutation, 61 (Additional file 1: Figure S1). Patients had from 1 to 16 alterations. Only 1 alteration was identified in 708 patients.

**Table 1:** Baseline characteristics of 1307 patients who had molecular alterations

Characteristic	*N* (%)	Matched therapy (%)	Non-matched therapy (%)	*p*
		*N* = 711	*N* = 596	
Age, years				.38
< 60	708 (54.2)	393 (55.3)	315 (52.9)	
≥ 60	599 (45.8)	318 (44.7)	281 (47.1)	
Sex				.67
Female	802 (61.4)	440 (61.9)	362 (6.7)	
Male	505 (38.6)	271 (38.1)	234 (39.3)	
Number of prior therapies				.41
≤ 3	637 (48.7)	354 (49.8)	283 (47.5)	
> 3	670 (51.3)	357 (5.2)	313 (52.5)	
Performance status				.27
0–1	1211 (92.7)	664 (93.4)	547 (91.8)	
> 1	96 (7.3)	47 (6.6)	49 (8.2)	
Platelet count, × 10^9^/L				.815
≤ 440	1254 (95.9)	683 (96.1)	571 (95.8)	
> 440	53 (4.1)	28 (3.9)	25 (4.2)	
Number of metastatic sites				.61
0-2	867 (66.3)	476 (66.9)	391 (65.6)	
> 2	440 (33.7)	235 (33.1)	205 (34.4)	
Liver metastases				.03
No	839 (64.2)	475 (66.8)	364 (61.1)	
Yes	468 (35.8)	236 (33.2)	232 (38.9)	
Lactate dehydrogenase, IU/L				< .001
≤ 618	856 (65.5)	499 (7.2)	357 (59.9)	
> 618	451 (34.5)	212 (29.8)	239 (4.1)	
Albumin, g/dL				.16
< 3.5	1185 (9.7)	652 (91.7)	533 (89.4)	
≥ 3.5	122 (9.3)	59 (8.3)	63 (1.6)	
Tumor type				N/A*
Breast	177 (13.5)	120 (16.9)	57 (9.6)	
Colorectal	238 (18.2)	90 (12.7)	148 (24.8)	
Endometrial	55 (4.2)	40 (5.6)	15 (2.5)	
Gastrointestinal, other	79 (6.0)	33 (4.6)	46 (7.7)	
Genitourinary, other	35 (2.7)	17 (2.4)	18 (3.0)	
Gynecological, other	67 (5.1)	35 (4.9)	32 (5.4)	
Head and neck	69 (5.3)	36 (5.1)	33 (5.5)	
Renal	12 (.9)	6 (.8)	6 (.8)	
Lung	114 (8.7)	71 (1.0)	43 (7.2)	
Melanoma	155 (11.9)	101 (14.2)	54 (9.1)	
Other	55 (4.2)	33 (4.6)	22 (3.7)	
Ovarian	132 (1.1)	59 (8.3)	73 (12.3)	
Pancreatic	25 (1.9)	7 (1.0)	18 (3.0)	
Sarcoma	33 (2.5)	19 (2.7)	14 (2.4)	
Thyroid	61 (4.7)	44 (6.2)	17 (2.9)	

*The distribution of tumor types is imbalanced between the two treatment groups; therefore, the *p* value is non-applicable

### Treatment

Of the 1307 patients treated, 711 (54.4%) received matched therapy and 596 (45.6%) had non-matched therapy.

### Response to therapy

Overall, 689 of 711 patients who were treated with matched therapy and 567 of 596 who were treated with non-matched therapy were evaluable for response. The remaining patients did not have imaging studies for restaging or withdrew consent prior to the first response assessment. Of the 689 evaluable patients in the matched group, 19 (2.8%) had a complete response (CR), 94 (13.6%) had a partial response (PR), and 130 (18.9%) had stable disease (SD) for ≥ 6 months.

Of the 567 evaluable patients in the non-matched therapy group, 3 (.5%) had a CR, 28 (4.9%) had a PR, and 84 (14.8%) had SD ≥ 6 months. The respective disease control rates were 35.3% and 20.3% (*p* < .001). Response by patient baseline characteristics is listed in Additional file 1: Table S2 (univariate analysis). Factors associated with higher rates of CR+PR+SD ≥ 6 months were performance status (0-1), number of metastatic sites (0-2), absence of liver metastases, and normal levels of albumin and lactate dehydrogenase (LDH). In multivariate analysis, factors that independently correlated with worse clinical benefit rates were non-matched therapy (*p* = .01), PI3K/AKT/mTOR pathway abnormalities (*p* = .02), liver metastases (*p* < .001), and LDH levels > the upper limit of normal (ULN) (*p* = .01) (Table 2).

**Table 2:** Clinical benefit and progression-free survival: multivariate analyses in patients with molecular alterations

Clinical Benefit (CR + PR + SD ≥ 6 months), evaluable for response (*N* = 1256)			
Risk Factor (vs. other)	OR	95% CI	*p*
PI3K/AKT/mTOR alterations	.73	.53–1.02	.06
Liver metastases	.54	.39–.76	< .001
LDH > ULN	.61	.43–.86	.004
Performance status > 1	.50	.24–1.02	.06
Albumin < ULN	.68	.37–1.25	.21
Type of therapy added			
Non-matched therapy	.67	.49–.90	.01
PI3K/AKT/mTOR alterations	.67	.48–.94	.02
Liver metastases	.55	.39–.77	< .001
LDH > ULN	.63	.45–.89	.01
Performance status > 1	.51	.25–1.05	.07
Albumin < ULN	.69	.97–1.27	.23
Progression-Free Survival (*n* = 1307)			
Risk Factor (vs. other)	HR	95% CI	*p*
PI3K/AKT/mTOR alterations	1.11	.98–1.27	.09
Liver metastases	1.45	1.27–1.64	< .001
LDH > ULN	1.50	1.31–1.70	< .001
Performance status > 1	1.57	1.24–1.99	< .001
Albumin < ULN	1.35	1.09–1.67	.01
Platelets > ULN	1.14	.85–1.53	.39
Age ≥ 60 years	.97	.86–1.09	.57
Type of therapy added			
Non-matched therapy	1.39	1.23–1.58	< .001
PI3K/AKT/mTOR alterations	1.16	1.02–1.32	.02
Liver metastases	1.43	1.26–1.62	< .001
LDH > ULN	1.44	1.26–1.64	< .001
Performance status > 1	1.54	1.22–1.95	< .001
Albumin < ULN	1.31	1.06–1.62	.01
Platelet count > ULN	1.09	.81–1.46	.59
Age ≥ 60 years	.95	.84–1.07	.37

*Abbreviations*: *CI* confidence interval, *CR* complete response, *HR* hazard ratio, *LDH* lactate dehydrogenase, *PR* partial response, *SD* stable disease, *ULN* upper limit of normal

### Progression-free survival

**Fig. 2:**
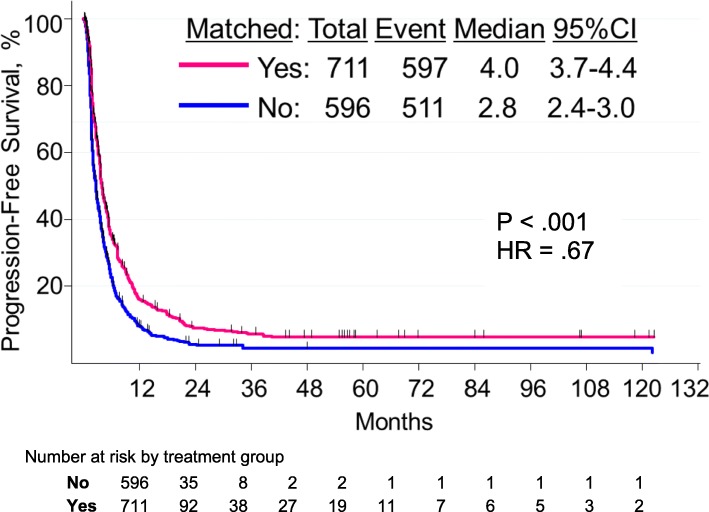
Progression-free survival by type of therapy

The median PFS duration of the 711 patients in the matched group was 4 months (95% confidence interval [CI], 3.7–4.4 months) compared with 2.8 months (95% CI, 2.4–3.0 months) in the 596 patients in the non-matched therapy group (hazard ratio [HR] = .67; *p* < .001) (Fig. 2). Univariate analyses of all patients (*n* = 1307) and of the matched (*n* = 711) and non-matched (*n* = 596) groups are shown in Additional file 1: Table S3. In univariate analysis (*n* = 1307), baseline characteristics associated with shorter PFS were performance status > 1, metastatic sites > 2, liver metastases, LDH levels > ULN, and albumin levels < ULN; PI3K/AKT/mTOR pathway abnormalities were associated with a trend towards shorter PFS. In patients treated with matched therapy, the PFS rates by pathway are shown in Additional file 1: Figure S2.

In multivariate analysis, factors that independently correlated with shorter PFS were performance status > 1 (*p* < .001), liver metastases (*p* < .001), albumin levels < ULN (*p* = .01), and LDH levels > ULN (*p* < .001) (Table 2). When type of therapy was added to the model, non-matched therapy and PI3K/AKT/mTOR pathway abnormalities were also independent factors predicting shorter PFS (*p* < .001 and *p* = .02, respectively) (Table 2).

### Overall survival

**Fig. 3:**
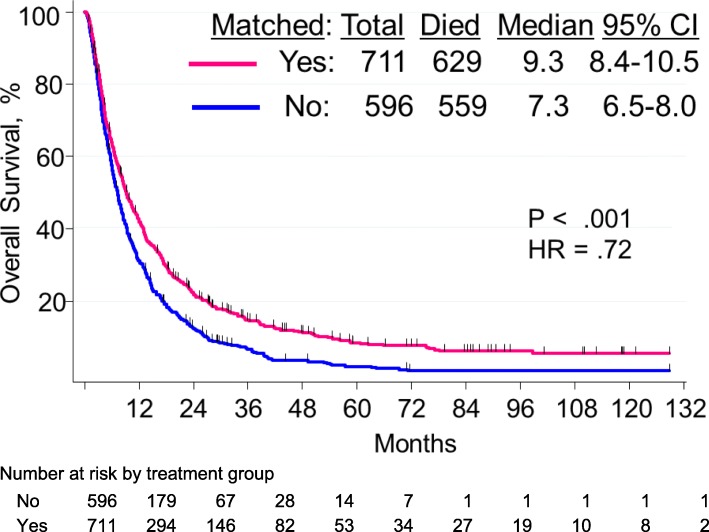
Overall survival by type of therapy

The median OS duration of the matched therapy group (*n* = 711) was 9.3 months (95% CI, 8.4–1.5 months), compared with 7.3 months (95% CI, 6.5–8.0 months) for the non-matched therapy group (*n* = 596). The 3-year OS rate was 15% in the matched therapy group compared with 7% in the non-matched group. The 10-year OS rates were 6% vs. 1%, respectively (HR = .72; *p* < .001) (Fig. 3). In univariate analysis of the training patient set (903 of 1307 patients), pretreatment factors associated with shorter survival were PI3K/Akt/mTOR alterations, age ≥ 60 years, performance status > 1, liver metastases, platelet count > ULN, LDH levels > ULN, albumin levels <ULN, and metastatic sites > 2 (Additional file 1: Table S4). The median OS of patients with PI3K/AKT/mTOR pathway alterations treated with matched therapy was 6.5 months, compared to 10.9, 12.6, and 11.6 months, respectively, for patients treated with MAPK signaling, tyrosine kinase, and hormone inhibitors (*p* < .001; Additional file 1: Figure S3). Multivariate analyses of the training and validation patient sets, as well as data for all patients are shown in Table 3. Independent factors associated with shorter OS (multivariate analysis) were performance status > 1 (*p* < .001), liver metastases (*p* < .001), LDH levels > upper limit of normal (*p* < .001), PI3K/AKT/mTOR pathway abnormalities (*p* < .001), and non-matched therapy (*p* < .001).

**Table 3:** Multivariate analyses for overall survival and scoring system

Factors independently prognostic of shorter overall survival	HR	95%CI		*p* value
Training patient set (*n* = 903)				
Non-matched therapy	1.20	1.04	1.38	0.01
Performance status > 1	2.52	1.94	3.28	< 0.001
Liver metastases	1.52	1.31	1.77	< 0.001
LDH > ULN	1.66	1.42	1.93	< 0.001
PI3K/AKT/mTOR pathway alterations	1.18	1.01	1.37	0.03
Validation patient set (*n* = 404)				
Non-matched therapy	1.59	1.29	1.96	< 0.001
Performance status > 1	2.21	1.53	3.20	< 0.001
Liver metastases	1.35	1.07	1.69	0.01
LDH > ULN	1.70	1.36	2.12	< 0.001
PI3K/AKT/mTOR pathway alterations	1.43	1.14	1.80	0.002
All patients (*N* = 1307)				
Non-matched therapy	1.32	1.17	1.48	< 0.001
Performance status > 1	2.38	1.93	2.95	< 0.001
Liver metastases	1.44	1.28	1.63	< 0.001
LDH > ULN	1.66	1.46	1.88	< 0.001
PI3K/AKT/mTOR pathway alterations	1.25	1.10	1.42	< 0.001
Scoring system for survival model				
Factors	HR	Score		
Matched therapy				
Yes	1.00	0		
No	1.32	1		
Performance status				
0–1	1.00	0		
2–3	2.38	2		
Liver metastases				
No	1.00	0		
Yes	1.44	1		
LDH > ULN				
No	1.00	0		
Yes	1.66	1		
PI3K/AKT/mTOR pathway alterations				
No	1.00	0		
Yes	1.25	1		

*Abbreviations*: *CI* confidence interval, *HR* hazard ratio, *LDH* lactate dehydrogenase, *ULN* upper limit of normal

Clinical outcomes by type of treatment, taking into consideration the tumor type and patient age and sex, are shown in Additional file 1: Table S5. Tumor types that were associated with better outcomes with matched therapy compared to non-matched therapy were breast cancer, colorectal cancer, gynecological tumors, lung cancer, melanoma, pancreatic cancer, sarcoma, thyroid cancer, and other tumors. Matched therapy was not associated with better outcomes compared to non-matched therapy in the remaining tumor types (ovarian, renal, head and neck, endometrial, other gastrointestinal, and other genitourinary cancers); however, limited numbers of patients may have precluded robust statistical analysis (Additional file 1: Table S5). Matched therapy was associated with better outcomes compared to non-matched therapy in both males and females and in both age groups (< 60 years and ≥ 60 years).

### Independent prognostic factors and prognostic score

**Fig. 4:**
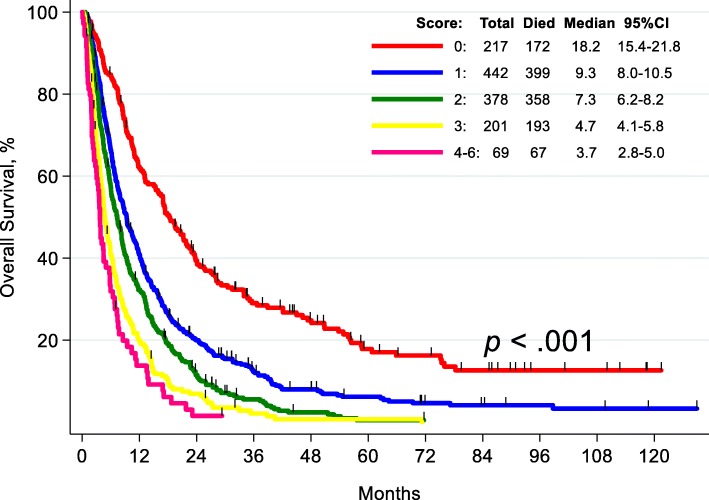
Overall survival by prognostic score. The five risk factors correlating independently with shorter survival were non-matched therapy (1 point), performance status > 1 (2 points), liver metastases (1 point), LDH levels > upper limit of normal (1 point), and PI3K/Akt/mTOR pathway alterations (1 point)

The five factors that remained independently significant in the multivariate analysis for OS of the 1307 treated patients (Table 3) were used to develop a prognostic score to predict an individual patient’s risk of death. On the basis of the hazard ratio of each factor (Table 3), a score of 1 was assigned to non-matched therapy, liver metastases, LDH > the upper limit of normal, and PI3k/AKT/mTOR pathway alterations and a score of 2 was assigned to performance status > 1. The risk of death was characterized by summing the score for each risk factor. We combined scores with similar risk (4–6) into a single category. The median OS duration of patients with 0 risk factors was 18.2 months; 1 risk factor, 9.3 months; 2 risk factors, 7.3 months; 3 risk factors, 4.7 months, and 4–6 risk factors, 3.7 months (Fig. 4).

## Discussion

We report on 1307 (37.5% of 3487 molecularly profiled) patients who had targetable molecular alterations and received treatment, including long-term follow-up. Of 1307 patients with ≥ 1 alteration, 54.4% received matched targeted therapy and 45.6% received non-matched therapy. The objective response rates were 16.4% and 5.4%, respectively (*p* < .0001). The overall disease control rates (objective response plus SD ≥ 6 months) were 35.3% and 20.3%, respectively (*p* < .001). The respective median PFS durations were 4.0 and 2.8 months (*p* < .0001), and the respective OS durations were 9.3 months and 7.3 months (*p* < .0001). The 3-year OS rate was 15% in the matched targeted group compared to 7% in the non-matched group. The 10-year OS rates were 6% and 1%, respectively. This was, to our knowledge, the first large precision medicine study across tumor types in patients who were referred for phase I clinical trials, and consequently, it has the longest follow-up.

In multivariate analysis, matched therapy was an independent factor predicting higher rates of clinical benefit, and longer PFS and OS. Our study demonstrated that PI3K/AKT/mTOR pathway alterations were associated with shorter OS compared to other alterations, probably because in advanced metastatic cancer, investigational agents targeting this pathway are not as effective as those targeting other pathways; there are escape mechanisms; or there is intrinsic resistance.

We also developed a prognostic score for OS. This score, which includes 5 variables (Fig. 4), can provide specific information that can be exploited to estimate OS for patients treated in clinical trials, especially in the phase I setting. Patients with a score of 0 had a median survival duration of 18.2 months, while patients with a score of 4 to 6 had a median OS duration of only 3.7 months. This prognostic score overlapped with many, but not all, of the variables in the Royal Marsden Hospital score and the previously published MD Anderson score [9, 10]. Importantly, unlike the previous scoring systems, this is the first score that incorporates molecular pathway analysis, as PI3K/Akt/mTOR pathway alterations were independently associated with shorter survival.

The proportion of patients who had targetable alterations in the current study is lower than estimates of targetable alterations in other publications, perhaps because the latter studies included only patients whose tumors were profiled by next-generation sequencing panels (≥ 200 genes) [11, 12].

Since the first IMPACT study was initiated, several targeted agents have been approved by the FDA on the basis of their superior outcomes compared to standard treatments in patients with specific tumor types and molecular alterations; examples include vemurafenib, crizotinib, dabrafenib, and trametinib [13–16]. Von Hoff et al. found that 98% of patients with cancer had a tumor alteration, including overexpression of genes in the tumor compared to the control organ tissue [17]. Other investigators demonstrated that targeted agents such as BYL719 (PI3Kα inhibitor), GDC-0032 (β isoform-sparing PI3K inhibitor), and AZD5363 (AKT1, 2, and 3 inhibitor) [18] were associated with benefit in patients with alterations in the PI3K/AKT/mTOR pathway [18–20]. We have previously reported that the use of PI3K/AKT/mTOR inhibitors is associated with encouraging results in patients with alterations in this pathway [21, 22].

Our results are consistent with data reported by other investigators. In a multicenter study of 1007 patients with metastatic lung adenocarcinoma, 64% had an oncogenic driver [23]; of the patients with oncogenic drivers, those who received targeted therapy had longer survival than those who did not receive targeted therapy (median, 3.5 years vs. 2.4 years; HR = .69, *p* = .006) [23]. In the MOSCATO (Molecular profiling in Cancer for Treatment Optimization) study, 19% of patients with metastatic cancer were treated according to their molecular profiles (objective response rate, 15%) [24].

In SHIVA, a randomized study for advanced cancer, no difference was noted in PFS between genomically matched therapy and conventional therapy [25]. That study was limited, primarily, by the use of a predefined algorithm for matched therapy compared to physicians’ choice of treatment for the control arm [26].

The key limitations to precision medicine include the overabundance of tests, which are continually increasing; the small number of patients with specific alterations treated with matched therapy from which to draw robust conclusions; the unavailability of drugs to treat some driver targets; and the complexity of tumor biology. Randomized studies with adaptive design that investigate various alterations and modeling are assessing the usefulness of tumor testing to guide optimal matched targeted therapy for individual patients [27].

The main strengths of the current study are: (1) it has the longest follow-up among studies in precision medicine across tumor types and (2) it included molecular pathway abnormalities in a prognostic model to predict the expected OS of individual patients who are being considered for clinical trials.

Although the data presented herein are extensive, the current study has limitations, including the retrospective analysis of outcomes of patients who were prospectively molecularly profiled to select therapy (non-randomized), the inclusion of multiple tumor types (given the nature of our program), the relatively small number of alterations tested during the study period, and the variability of available clinical trials depending on time of treatment. Given that the current study was initiated in 2007, when DNA testing was technologically limited and allowed testing of only a few genes, caution is warranted in the interpretation of the results. It is plausible that multiple other alterations coexisted with the identified ones, as well as other mechanisms of carcinogenesis. Other challenges included the lack of targeted combination therapies that are effective against multiple alterations and the presence of other unknown mechanisms involved in carcinogenesis that could not be examined and could not be inhibited with the use of available therapies.

We are conducting a phase II randomized study evaluating molecular profiling and targeted therapy in metastatic cancer (IMPACT 2; NCT02152254) to address the weaknesses of our first IMPACT study [4, 5]. The primary objective of IMPACT 2 is to compare PFS in patients treated with targeted therapy selected on the basis of tumor molecular analysis with PFS in those whose treatment was not selected on the basis of molecular analysis. The American Society of Clinical Oncology is enrolling patients in the Targeted Agent and Profiling Utilization Registry (TAPUR) study (non-randomized). The objective of TAPUR is to assess the efficacy and toxicity of FDA-approved targeted anticancer agents in patients with advanced cancer with a potentially actionable genomic alteration. These and other ongoing trials offer treatment options to patients with advanced cancer and hold the promise of providing data to accelerate the implementation of precision medicine.

## Summary

In conclusion, our data demonstrate that matched targeted therapy is associated with superior rates of objective response, PFS, and long-term OS compared to non-matched therapy. The 3-year OS rate was 15% in the matched targeted group compared to 7% in the non-matched group, and the 10-year OS rates were 6% and 1%, respectively.

Independent factors predicting shorter OS in multivariate analysis were used to develop a prognostic score to predict an individual patient’s risk of death. These factors were non-matched therapy, liver metastases, LDH > the upper limit of normal, and PI3k/AKT/mTOR pathway alterations (score of 1 each), and performance status > 1 (score of 2). This prognostic model that includes molecular pathway abnormalities can be used to predict the expected OS of individual patients who are being considered for clinical trials.

Advances in technology and bioinformatics to identify driver molecular alterations; evolution of the global assessment of immune mechanisms and proteomic, transcriptomic, and epigenetic changes in individual patient tumor pathogenesis; and innovative, carefully designed clinical trials are expected to improve the implementation of precision medicine.

## Conclusions

- Analysis of our data demonstrated that in patients with metastatic cancer, matched targeted therapy is associated with superior rates of objective response, PFS, and long-term OS compared to non-matched therapy.
- This was the first large precision medicine study across tumor types in patients who were referred for phase I clinical trials, and consequently, it has the longest follow-up. The 3-year overall survival rate was 15% in the matched group compared to 7% in the non-matched group and the 10-year overall survival rates were 6% vs. 1%, respectively.
- Matched therapy was an independent factor predicting longer survival in multivariate analysis.
- *PI3K/Akt/mTOR* pathway abnormalities were associated with inferior outcomes compared to other alterations.
- Independent factors predicting shorter OS in multivariate analysis were used to develop a prognostic score to predict an individual patient’s risk of death. These factors were non-matched therapy, liver metastases, LDH greater than the upper limit of normal, and PI3k/AKT/mTOR pathway alterations (score of 1 each) and performance status greater than 1 (score of 2). This prognostic model that includes molecular pathway abnormalities can be used to predict the expected OS of individual patients who are being considered for clinical trials.
- Implementation of precision medicine will dramatically improve the outcomes of patients with cancer.

## Additional file

**Additional file 1: Table S1**. Molecular pathways and targets. **Table S2**. Univariate analysis: response by baseline characteristics of evaluable patients who had molecular alterations. **Table S3**. Univariate analysis: progression-free survival by baseline characteristics of 1,307 patients. **Table S4**. Univariate analysis of overall survival using the training data (~70% of the original data), N=903. **Table S5**. Univariate analysis of clinical benefit, progression-free survival (PFS), and overall survival (OS) by tumor type, sex, and age group. **Figure S1**. Bars indicate the number of patients whose tumors had a particular molecular aberration. In patients with multiple molecular alterations, each alteration was counted separately. **Figure S2**. Progression-free survival by pathway in patients treated with matched therapy. **Figure S3**. Overall survival by pathway in all treated patients.

## Data Availability

The data and material are available from the corresponding author upon request and following institutional guidelines.
