# Supplementary material for: Long-term overall survival and prognostic score predicting survival: the IMPACT study in precision medicine
Source: J Hematol Oncol. 2019 Dec 30;12:145. doi: 10.1186/s13045-019-0835-1 (PMC6937824; doi:10.1186/s13045-019-0835-1)
Supplement: Supplementary file 1 — Additional file 1: Table S1. Molecular pathways and targets. Table S2. Univariate analysis: response by baseline characteristics of evaluable patients who had molecular alterations. Table S3. Univariate analysis: progression-free survival by baseline characteristics of 1,307 patients. Table S4. Univariate analysis of overall survival using the training data (~70% of the original data), N=903. Table S5. Univariate analysis of clinical benefit, progression-free survival (PFS), and overall survival (OS) by tumor type, sex, and age group. Figure S1. Bars indicate the number of patients whose tumors had a particular molecular aberration. In patients with multiple molecular alterations, each alteration was counted separately. Figure S2. Progression-free survival by pathway in patients treated with matched therapy. Figure S3. Overall survival by pathway in all treated patients. [file 13045_2019_835_MOESM1_ESM.docx]

**Additional file 1: Table S1. Molecular pathways and targets**

| **Tyrosine kinases** | FGFR1, FGFR2, FGFR3, FGFR4, EGFR, ERBB2, ALK, KIT, DDR2, KDR, PDGFRA, PDGFRB, FLT3, FLT4, JAK2, SRC, HER2, RET |
| --- | --- |
| **PI3K/Akt/mTOR pathway** | PIK3CA, PIK3R1, PTEN, AKT2, STK11, TSC1, TSC2, RPTOR, MTOR, RICTOR, NF2 |
| **Cell cycle–associated genes** | CDKN2A/B, CDKN2A, CDKN2C, CCND1, CCND2, CCNE1, CDK6, RB1 |
| **TP53/ Tumor suppressor/ Apoptosis–associated genes** | MDM2, ATM, TP53, PTCH1, VHL, BCL2, BAD |
| **MAPK signaling** | NF1, KRAS, NRAS, BRAF, GNAQ |
| **Hormone pathway** | ER, PR, AR |
| **DNA repair pathway** | BRCA |

**Additional file 1: Table S2:** Univariate analysis: response by baseline characteristics of evaluable patients who had molecular alterations

| Characteristic | Matched | CR+PR+  SD≥6 months, % | P | Non-Matched | CR+PR+  SD≥6 months, % | P | Total | CR+PR+  SD≥6 months, % | P |
| --- | --- | --- | --- | --- | --- | --- | --- | --- | --- |
|  | N=689 |  |  | N=567 |  |  | N=1256 |  |  |
| Age, years |  |  | .73 |  |  | .33 |  |  | .42 |
| <60 | 383 | 21.15 |  | 301 | 13.95 |  | 684 | 17.98 |  |
| ≥60 | 306 | 22.22 |  | 266 | 16.92 |  | 572 | 19.76 |  |
|  |  |  |  |  |  |  |  |  |  |
| Sex |  |  | .17 |  |  | .75 |  |  | .35 |
| Female | 424 | 23.35 |  | 341 | 14.96 |  | 765 | 19.61 |  |
| Male | 265 | 18.87 |  | 226 | 15.93 |  | 491 | 17.52 |  |
|  |  |  |  |  |  |  |  |  |  |
| Number of prior therapies |  |  | .86 |  |  | .002 |  |  | .06 |
| ≤3 | 342 | 21.35 |  | 271 | 2.30 |  | 613 | 2.88 |  |
| >3 | 347 | 21.90 |  | 296 | 1.81 |  | 643 | 16.80 |  |
|  |  |  |  |  |  |  |  |  |  |
| Performance status |  |  | .01 |  |  | .65 |  |  | .03 |
| 0-1 | 645 | 22.64 |  | 521 | 15.55 |  | 1166 | 19.47 |  |
| >1 | 44 | 6.82 |  | 46 | 13.04 |  | 90 | 1.00 |  |
|  |  |  |  |  |  |  |  |  |  |
| Platelet count,  x 10^9^/L |  |  | .02 |  |  | .18 |  |  | .35 |
| ≤440 | 662 | 22.36 |  | 543 | 14.92 |  | 1205 | 19.00 |  |
| >440 | 27 | 3.70 |  | 24 | 25.00 |  | 51 | 13.73 |  |
|  |  |  |  |  |  |  |  |  |  |
| Number of metastatic sites |  |  | .23 |  |  | .05 |  |  | .03 |
| 0-2 | 462 | 22.94 |  | 372 | 17.47 |  | 834 | 2.50 |  |
| >2 | 227 | 18.94 |  | 195 | 11.28 |  | 422 | 15.40 |  |
|  |  |  |  |  |  |  |  |  |  |
| Liver metastases |  |  | .001 |  |  | .01 |  |  | <.001 |
| No | 461 | 25.38 |  | 347 | 18.44 |  | 808 | 22.40 |  |
| Yes | 228 | 14.04 |  | 220 | 1.45 |  | 448 | 12.28 |  |
|  |  |  |  |  |  |  |  |  |  |
| Lactate dehydrogenase, IU/L |  |  | .01 |  |  | .02 |  |  | <.001 |
| ≤618 | 486 | 24.49 |  | 342 | 18.13 |  | 828 | 21.86 |  |
| >618 | 203 | 14.78 |  | 225 | 11.11 |  | 428 | 12.85 |  |
|  |  |  |  |  |  |  |  |  |  |
| Albumin, g/dL |  |  | .08 |  |  | .27 |  |  | .03 |
| <3.5 | 633 | 22.43 |  | 509 | 15.91 |  | 1142 | 19.53 |  |
| ≥3.5 | 56 | 15.50 |  | 58 | 1.34 |  | 114 | 11.40 |  |
|  |  |  |  |  |  |  |  |  |  |
| Molecular alterations |  |  | .01 |  |  | .06 |  |  | .14 |
| PI3K/AKT/mTOR pathway | 258 | 14.73 |  | 112 | 18.75 |  | 370 | 15.95 |  |
| MAPK signaling | 217 | 24.88 |  | 192 | 9.90 |  | 409 | 17.85 |  |
| Tyrosine kinases | 127 | 27.56 |  | 67 | 14.93 |  | 194 | 23.20 |  |
| Hormone and Others | 87 | 25.29 |  | 196 | 18.88 |  | 283 | 2.85 |  |
|  |  |  |  |  |  |  |  |  |  |
| Tumor type |  |  | N/A |  |  | N/A |  |  | N/A |
| Breast | 117 | 23.93 |  | 55 | 12.73 |  | 172 | 2.35 |  |
| Colorectal | 86 | 11.63 |  | 139 | 5.76 |  | 225 | 8.00 |  |
| Endometrial | 40 | 1.00 |  | 13 | 15.38 |  | 53 | 11.32 |  |
| Gastrointestinal, other | 31 | 22.58 |  | 45 | 17.78 |  | 76 | 19.74 |  |
| Genitourinary, other | 16 | 6.25 |  | 18 | 44.44 |  | 34 | 26.47 |  |
| Gynecological, other | 34 | 23.53 |  | 28 | 21.43 |  | 62 | 22.58 |  |
| Head and neck | 36 | 11.11 |  | 33 | 18.18 |  | 69 | 14.49 |  |
| Renal | 6 | .00 |  | 6 | 16.67 |  | 12 | 8.33 |  |
| Lung | 68 | 22.06 |  | 43 | 16.28 |  | 111 | 19.82 |  |
| Melanoma | 99 | 23.23 |  | 52 | 7.69 |  | 151 | 17.88 |  |
| Other | 33 | 27.27 |  | 19 | 15.79 |  | 52 | 23.08 |  |
| Ovarian | 56 | 19.64 |  | 68 | 2.59 |  | 124 | 2.16 |  |
| Pancreatic | 7 | 14.29 |  | 18 | 11.11 |  | 25 | 12.00 |  |
| Sarcoma | 18 | 33.33 |  | 14 | 28.57 |  | 32 | 31.25 |  |
| Thyroid | 42 | 52.38 |  | 16 | 43.75 |  | 58 | 5.00 |  |

* The distribution of tumor types is imbalanced between the two treatment groups; therefore, the p-value is non-informative.

**Additional file 1: Table S3**: Univariate analysis: progression-free survival by baseline characteristics of 1,307 patients

| Characteristic | Matched | Median PFS, months | P-value | Non-Matched | Median PFS, months | P-value | Total  Matched and Non-Matched | Median PFS, months | P |
| --- | --- | --- | --- | --- | --- | --- | --- | --- | --- |
|  | N=711 |  |  | N=596 |  |  | N=1307 |  |  |
| Age, years |  |  |  |  |  |  |  |  |  |
| <60 | 393 | 4.01 | .54 | 315 | 2.76 | .67 | 708 | 3.61 | .59 |
| ≤60 | 318 | 4.07 |  | 281 | 2.76 |  | 599 | 3.45 |  |
|  |  |  |  |  |  |  |  |  |  |
| Sex |  |  |  |  |  |  |  |  |  |
| Female | 440 | 4.37 | .89 | 362 | 2.89 | .70 | 802 | 3.65 | .66 |
| Male | 271 | 3.84 |  | 234 | 2.40 |  | 505 | 3.38 |  |
|  |  |  |  |  |  |  |  |  |  |
| Number of prior therapies |  |  |  |  |  |  |  |  |  |
| ≤3 | 354 | 3.75 | .44 | 283 | 2.76 | .12 | 637 | 3.52 | .78 |
| >3 | 357 | 4.17 |  | 313 | 2.76 |  | 670 | 3.52 |  |
|  |  |  |  |  |  |  |  |  |  |
| Performance status |  |  |  |  |  |  |  |  |  |
| 0-1 | 664 | 4.17 | <.0001 | 547 | 2.76 | .14 | 1211 | 3.65 | <.0001 |
| >1 | 47 | 2.30 |  | 49 | 1.94 |  | 96 | 2.00 |  |
|  |  |  |  |  |  |  |  |  |  |
| Platelet count,  x 10^9^/L |  |  |  |  |  |  |  |  |  |
| ≤440 | 683 | 4.14 | .004 | 571 | 2.76 | .52 | 1254 | 3.61 | .10 |
| >440 | 28 | 2.76 |  | 25 | 2.50 |  | 53 | 2.76 |  |
|  |  |  |  |  |  |  |  |  |  |
| Number of metastatic sites |  |  |  |  |  |  |  |  |  |
| 0-2 | 476 | 4.24 | .024 | 391 | 3.09 | .001 | 867 | 3.68 | <.0001 |
| >2 | 235 | 3.68 |  | 205 | 2.00 |  | 440 | 2.96 |  |
|  |  |  |  |  |  |  |  |  |  |
| Liver metastases |  |  |  |  |  |  |  |  |  |
| No | 475 | 4.83 | <.0001 | 364 | 3.15 | <.0001 | 839 | 4.14 | <.0001 |
| Yes | 236 | 3.12 |  | 232 | 2.07 |  | 468 | 2.56 |  |
|  |  |  |  |  |  |  |  |  |  |
| Lactate dehydrogenase, IU/L |  |  |  |  |  |  |  |  |  |
| ≤618 | 499 | 4.37 | <.0001 | 357 | 3.25 | <.0001 | 856 | 3.91 | <.0001 |
| >618 | 212 | 3.06 |  | 239 | 2.07 |  | 451 | 2.33 |  |
|  |  |  |  |  |  |  |  |  |  |
| Albumin, g/dL |  |  |  |  |  |  |  |  |  |
| <3.5 | 652 | 4.21 | <.0001 | 533 | 2.76 | .07 | 1185 | 3.68 | <.0001 |
| ≥3.5 | 59 | 1.94 |  | 63 | 2.07 |  | 122 | 2.07 |  |
|  |  |  |  |  |  |  |  |  |  |
| Molecular alterations |  |  |  |  |  |  |  |  |  |
| PI3K/AKT/mTOR pathway | 268 | 3.48 | .001 | 117 | 2.56 | .005 | 385 | 3.25 | .06 |
| MAPK signaling | 225 | 4.60 |  | 204 | 2.04 |  | 429 | 3.61 |  |
| Tyrosine kinases | 128 | 5.03 |  | 69 | 3.32 |  | 197 | 4.37 |  |
| Hormone and Others | 90 | 4.53 |  | 206 | 3.52 |  | 296 | 3.68 |  |
|  |  |  |  |  |  |  |  |  |  |
| Tumor type |  |  |  |  |  |  |  |  |  |
| Breast | 120 | 4.34 | N/A | 57 | 2.56 | N/A | 177 | 3.45 | N/A |
| Colorectal | 90 | 2.53 |  | 148 | 2.07 |  | 238 | 2.27 |  |
| Endometrial | 40 | 3.19 |  | 15 | 4.17 |  | 55 | 4.04 |  |
| Gastrointestinal, other | 33 | 2.99 |  | 46 | 2.76 |  | 79 | 2.76 |  |
| Genitourinary, other | 17 | 2.27 |  | 18 | 6.47 |  | 35 | 3.68 |  |
| Gynecological, other | 35 | 4.34 |  | 32 | 2.50 |  | 67 | 3.29 |  |
| Head and neck | 36 | 3.12 |  | 33 | 4.01 |  | 69 | 3.75 |  |
| Renal | 6 | 4.76 |  | 6 | 1.84 |  | 12 | 2.79 |  |
| Lung | 71 | 3.68 |  | 43 | 2.76 |  | 114 | 3.15 |  |
| Melanoma | 101 | 5.16 |  | 54 | 2.30 |  | 155 | 4.14 |  |
| Other | 33 | 5.29 |  | 22 | 2.40 |  | 55 | 4.04 |  |
| Ovarian | 59 | 4.83 |  | 73 | 5.19 |  | 132 | 4.83 |  |
| Pancreatic | 7 | 4.34 |  | 18 | 2.76 |  | 25 | 2.86 |  |
| Sarcoma | 19 | 7.29 |  | 14 | 2.37 |  | 33 | 4.60 |  |
| Thyroid | 44 | 21.39 |  | 17 | 6.05 |  | 61 | 16.95 |  |

* The distribution of tumor types is imbalanced between the two treatment groups; therefore, a p-value would not be informative.

**Additional file 1: Table S4**: **Univariate analysis of overall survival using the training data (~70% of the original data), N=903**

|  | N | Events | Median | log-rank | HR | 95% CI for HR | p-value |
| --- | --- | --- | --- | --- | --- | --- | --- |
| Sex |  |  |  |  |  |  |  |
| F | 552 | 492 | 8.57 | 0.332 |  |  |  |
| M | 351 | 323 | 7.56 |  | 1.07 | (0.93-1.23) | 0.333 |
| ECOG |  |  |  |  |  |  |  |
| 0-1 | 839 | 752 | 8.64 | <0.001 |  |  |  |
| 2-3 | 64 | 63 | 3.02 |  | 2.53 | (1.95-3.28) | <0.001 |
| Liver metastases |  |  |  |  |  |  |  |
| No | 560 | 492 | 10.35 | <0.001 |  |  |  |
| Yes | 343 | 323 | 5.88 |  | 1.71 | (1.48-1.98) | <0.001 |
| Age≥60 |  |  |  |  |  |  |  |
| No | 488 | 426 | 8.71 | 0.004 |  |  |  |
| Yes | 415 | 389 | 7.75 |  | 1.23 | (1.07-1.41) | 0.004 |
| Albumin<3.5 g/dL |  |  |  |  |  |  |  |
| No | 824 | 742 | 8.64 | <0.001 |  |  |  |
| Yes | 79 | 73 | 3.52 |  | 1.81 | (1.42-2.30) | <0.001 |
| Number of prior therapies>3 |  |  |  |  |  |  |  |
| No | 439 | 386 | 8.41 | 0.520 |  |  |  |
| Yes | 464 | 429 | 8.21 |  | 1.05 | (0.91-1.20) | 0.520 |
| Platelet count x 10^9/L |  |  |  |  |  |  |  |
| ≤440 | 862 | 775 | 8.44 | 0.006 |  |  |  |
| >440 | 41 | 40 | 5.09 |  | 1.55 | (1.13-2.14) | 0.007 |
| Number of metastatic sites>2 |  |  |  |  |  |  |  |
| No | 597 | 533 | 9.49 | <0.001 |  |  |  |
| Yes | 306 | 282 | 6.24 |  | 1.47 | (1.27-1.70) | <0.001 |
| Lactate dehydrogenase>/=618 IU/L |  |  |  |  |  |  |  |
| No | 590 | 517 | 10.38 | <0.001 |  |  |  |
| Yes | 313 | 298 | 5.52 |  | 1.89 | (1.64-2.19) | <0.001 |
| PI3K versus others |  |  |  |  |  |  |  |
| No | 627 | 565 | 8.67 | 0.051 |  |  |  |
| Yes | 276 | 250 | 7.10 |  | 1.16 | (1.00-1.35) | 0.051 |
| Matched therapy |  |  |  |  |  |  |  |
| No | 405 | 375 | 7.36 | <0.001 |  |  |  |
| Yes | 498 | 440 | 8.97 |  | 0.75 | (0.65-0.86) | <0.001 |

**Additional file 1: Table S5. Univariate analysis of clinical benefit, progression-free survival (PFS), and overall survival (OS) by tumor type, sex, and age group.**

|  |  | Type of Therapy | | | |  |  |  |  |
| --- | --- | --- | --- | --- | --- | --- | --- | --- | --- |
|  |  | Matched | | Non-Matched | |  | Ratio* | 95% CI | p-value |
|  |  | N |  | N |  |  |  |  |  |
| **Tumor type** |  |  |  |  |  |  |  |  |  |
| Breast |  |  |  |  |  |  |  |  |  |
| CR+PR +SD≥6 months | % | 117 | 23.93 | 55 | 12.73 | OR | 2.16 | (0.88-5.30) | 0.094 |
| PFS | Median | 120 | 4.34 | 57 | 2.56 | HR | 0.56 | (0.40-0.79) | 0.001 |
| OS | Median | 120 | 8.44 | 57 | 6.57 | HR | 0.73 | (0.53-1.02) | 0.066 |
| CRC |  |  |  |  |  |  |  |  |  |
| CR+PR +SD≥6 months | % | 86 | 11.63 | 139 | 5.76 | OR | 2.15 | (0.82-5.69) | 0.122 |
| PFS | Median | 90 | 2.53 | 148 | 2.07 | HR | 0.80 | (0.60-1.07) | 0.133 |
| OS | Median | 90 | 6.47 | 148 | 7.29 | HR | 0.93 | (0.71-1.22) | 0.595 |
| GYN other |  |  |  |  |  |  |  |  |  |
| CR+PR +SD≥6 months | % | 34 | 23.53 | 28 | 21.43 | OR | 1.13 | (0.34-3.75) | 0.844 |
| PFS | Median | 35 | 4.34 | 32 | 2.50 | HR | 0.71 | (0.42-1.22) | 0.218 |
| OS | Median | 35 | 8.64 | 32 | 6.31 | HR | 0.72 | (0.43-1.21) | 0.216 |
| Lung |  |  |  |  |  |  |  |  |  |
| CR+PR +SD≥6 months | % | 68 | 22.06 | 43 | 16.28 | OR | 1.46 | (0.54-3.93) | 0.458 |
| PFS | Median | 71 | 3.68 | 43 | 2.76 | HR | 0.80 | (0.53-1.20) | 0.275 |
| OS | Median | 71 | 6.44 | 43 | 6.70 | HR | 0.96 | (0.65-1.41) | 0.838 |
| Melanoma |  |  |  |  |  |  |  |  |  |
| CR+PR +SD≥6 months | % | 99 | 23.23 | 52 | 7.69 | OR | 3.63 | (1.18-11.15) | 0.024 |
| PFS | Median | 101 | 5.16 | 54 | 2.30 | HR | 0.43 | (0.30-0.62) | <0.001 |
| OS | Median | 101 | 10.45 | 54 | 5.49 | HR | 0.46 | (0.32-0.66) | <0.001 |
| Pancreatic |  |  |  |  |  |  |  |  |  |
| CR+PR +SD≥6 months | % | 7 | 14.29 | 18 | 11.11 | OR | 1.33 | (0.10-17.55) | 0.827 |
| PFS | Median | 7 | 4.34 | 18 | 2.76 | HR | 0.47 | (0.17-1.28) | 0.140 |
| OS | Median | 7 | 4.50 | 18 | 4.53 | HR | 0.90 | (0.35-2.33) | 0.822 |
| Sarcoma |  |  |  |  |  |  |  |  |  |
| CR+PR +SD≥6 months | % | 18 | 33.33 | 14 | 28.57 | OR | 1.25 | (0.27-5.71) | 0.773 |
| PFS | Median | 19 | 7.29 | 14 | 2.37 | HR | 0.52 | (0.24-1.15) | 0.106 |
| OS | Median | 19 | 17.35 | 14 | 8.80 | HR | 0.46 | (0.21-1.02) | 0.055 |
| Thyroid |  |  |  |  |  |  |  |  |  |
| CR+PR +SD≥6 months | % | 42 | 52.38 | 16 | 43.75 | OR | 1.41 | (0.44-4.51) | 0.558 |
| PFS | Median | 44 | 21.39 | 17 | 6.05 | HR | 0.32 | (0.17-0.62) | 0.001 |
| OS | Median | 44 | 44.94 | 17 | 20.93 | HR | 0.43 | (0.23-0.82) | 0.010 |
| Ovarian |  |  |  |  |  |  |  |  |  |
| CR+PR +SD≥6 months | % | 56 | 19.64 | 68 | 20.59 | OR | 0.94 | (0.39-2.28) | 0.896 |
| PFS | Median | 59 | 4.83 | 73 | 5.19 | HR | 0.86 | (0.58-1.28) | 0.454 |
| OS | Median | 59 | 11.20 | 73 | 8.61 | HR | 0.70 | (0.48-1.01) | 0.059 |
|  |  |  |  |  |  |  |  |  |  |
| **Tumor type** |  |  |  |  |  |  |  |  |  |
| Renal |  |  |  |  |  |  |  |  |  |
| CR+PR +SD≥6 months | % | 6 | 0.00 | 6 | 16.67 | OR | 1.00 | (1.00-1.00) | . |
| PFS | Median | 6 | 4.76 | 6 | 1.84 | HR | 0.36 | (0.09-1.55) | 0.171 |
| OS | Median | 6 | 7.49 | 6 | 6.11 | HR | 0.63 | (0.17-2.41) | 0.499 |
| Head & Neck |  |  |  |  |  |  |  |  |  |
| CR+PR +SD≥6 months | % | 36 | 11.11 | 33 | 18.18 | OR | 0.56 | (0.14-2.20) | 0.409 |
| PFS | Median | 36 | 3.12 | 33 | 4.01 | HR | 1.13 | (0.68-1.88) | 0.646 |
| OS | Median | 36 | 7.75 | 33 | 8.87 | HR | 1.15 | (0.70-1.88) | 0.575 |
| Endometrial |  |  |  |  |  |  |  |  |  |
| CR+PR +SD≥6 months | % | 40 | 10.00 | 13 | 15.38 | OR | 0.61 | (0.10-3.80) | 0.597 |
| PFS | Median | 40 | 3.19 | 15 | 4.17 | HR | 1.05 | (0.53-2.08) | 0.894 |
| OS | Median | 40 | 6.41 | 15 | 8.25 | HR | 1.07 | (0.57-2.01) | 0.837 |
| GI other |  |  |  |  |  |  |  |  |  |
| CR+PR +SD≥6 months | % | 31 | 22.58 | 45 | 17.78 | OR | 1.35 | (0.43-4.21) | 0.606 |
| PFS | Median | 33 | 2.99 | 46 | 2.76 | HR | 1.10 | (0.68-1.77) | 0.695 |
| OS | Median | 33 | 6.47 | 46 | 6.44 | HR | 1.24 | (0.77-1.99) | 0.383 |
| GU |  |  |  |  |  |  |  |  |  |
| CR+PR +SD≥6 months | % | 16 | 6.25 | 18 | 44.44 | OR | 0.08 | (0.01-0.77) | 0.029 |
| PFS | Median | 17 | 2.27 | 18 | 6.47 | HR | 2.67 | (1.18-6.03) | 0.019 |
| OS | Median | 17 | 6.57 | 18 | 13.24 | HR | 1.78 | (0.89-3.57) | 0.105 |
| Other |  |  |  |  |  |  |  |  |  |
| CR+PR +SD≥6 months | % | 33 | 27.27 | 19 | 15.79 | OR | 2.00 | (0.47-8.54) | 0.349 |
| PFS | Median | 33 | 5.29 | 22 | 2.40 | HR | 0.73 | (0.39-1.36) | 0.322 |
| OS | Median | 33 | 20.11 | 22 | 5.82 | HR | 0.49 | (0.27-0.89) | 0.020 |
|  |  |  |  |  |  |  |  |  |  |
| **Sex** |  |  |  |  |  |  |  |  |  |
| Female |  |  |  |  |  |  |  |  |  |
| CR+PR +SD≥6 months | % | 424 | 23.35 | 341 | 14.96 | OR | 1.73 | (1.19-2.52) | 0.004 |
| PFS | Median | 440 | 4.37 | 362 | 2.89 | HR | 0.67 | (0.58-0.78) | <0.001 |
| OS | Median | 440 | 9.49 | 362 | 7.33 | HR | 0.72 | (0.62-0.83) | <0.001 |
| Male |  |  |  |  |  |  |  |  |  |
| CR+PR +SD≥6 months | % | 265 | 18.87 | 226 | 15.93 | OR | 1.23 | (0.77-1.97) | 0.394 |
| PFS | Median | 271 | 3.84 | 234 | 2.40 | HR | 0.67 | (0.55-0.81) | <0.001 |
| OS | Median | 271 | 8.67 | 234 | 7.33 | HR | 0.73 | (0.60-0.87) | 0.001 |
|  |  |  |  |  |  |  |  |  |  |
| **Age, years** |  |  |  |  |  |  |  |  |  |
| <60 |  |  |  |  |  |  |  |  |  |
| CR+PR +SD≥6 months | % | 383 | 21.15 | 301 | 13.95 | OR | 1.65 | (1.10-2.49) | 0.016 |
| PFS | Median | 393 | 4.01 | 315 | 2.76 | HR | 0.68 | (0.58-0.80) | <0.001 |
| OS | Median | 393 | 9.59 | 315 | 7.52 | HR | 0.75 | (0.64-0.88) | <0.001 |
| ≥60 |  |  |  |  |  |  |  |  |  |
| CR+PR +SD≥6 months | % | 306 | 22.22 | 266 | 16.92 | OR | 1.40 | (0.92-2.13) | 0.113 |
| PFS | Median | 318 | 4.07 | 281 | 2.76 | HR | 0.66 | (0.55-0.79) | <0.001 |
| OS | Median | 318 | 8.84 | 281 | 6.60 | HR | 0.69 | (0.58-0.81) | <0.001 |

* OR (odds ratio) for clinical benefit compares matched therapy with non-matched therapy. OR > 1 indicates higher clinical benefit rates with matched therapy compared to non-matched therapy. OR<1 indicates lower clinical benefit rates with matched therapy compared to non-matched therapy.

HR (hazard ratio) for PFS and OS compares matched therapy with non-matched therapy. HR > 1 indicates higher risk of progression or death with matched therapy compared to non-matched therapy. HR<1 indicates lower risk of progression or death with matched therapy compared to non-matched therapy.

**Additional file 1: Figure S1:** Bars indicate the number of patients whose tumors had a particular molecular aberration. In patients with multiple molecular alterations, each alteration was counted separately.

**Additional file 1: Figure S2:** Progression-free survival by pathway in patients treated with matched therapy.

**Additional file 1: Figure S3:** Overall survival by pathway in all treated patients.

**Additional file 1: Figure S1**

One aberration only was identified in 708 patients; 2 alterations were found in 298 patients; 3 in 145; 4 in 60; 5 in 37; 6 in 24; 7 in 12; 8 in 12; 9 in 4; 10 in 4; 12 in 1; 14 in 1; and 16 in 1.

**Additional file 1: Figure S2**

**Additional file 1: Figure S3**
